# Pre-operative Axillary Staging in Breast Cancer: Implications and Outcome

**DOI:** 10.7759/cureus.102756

**Published:** 2026-01-31

**Authors:** Huma Irshad, Fiona Mavor, Shabana Avesi, Rebecca Bourdon-Pierre, Myat Win, Zatinahhayu Mohammad Isa

**Affiliations:** 1 Breast Surgery, Hereford County Hospital/Wye Valley Trust, Hereford, GBR; 2 Surgery, Warrington and Halton Teaching Hospitals Foundation Trust, Warrington, GBR; 3 Surgery, Leighton Hospital, Mid Cheshire Hospital Trust, Crewe, GBR; 4 General Surgery, Leighton Hospital, Mid Cheshire Hospital Trust, Crewe, GBR; 5 Radiology, Leighton Hospital, Mid Cheshire Hospital Trust, Crewe, GBR

**Keywords:** axillary assessment, breast, ncb, slnb, ultrasound

## Abstract

Introduction

Axillary ultrasonography (AUS) and US-guided needle core biopsy (NCB) are recommended for evaluating axillary nodal status in new breast cancer cases. Presence of axillary nodal metastasis significantly impacts five-year survival rates. This study evaluated the diagnostic accuracy of preoperative AUS and US‑guided NCB in patients with invasive breast cancer at Leighton Hospital, benchmarked against national NHS standards. The primary objective was to assess the sensitivity, specificity, positive predictive value (PPV), and negative predictive value (NPV) of AUS and NCB in detecting nodal metastasis.

Methodology

This retrospective study was conducted at the Breast Unit of Leighton Hospital, part of the Mid Cheshire Hospital Trust. The study population included all patients diagnosed and treated for breast cancer from January 1, 2022, to December 31, 2022. Patients presenting with locally advanced, metastatic, or recurrent breast cancer were excluded from the study. Ultrasound evaluations were performed using a high-frequency linear-array transducer (7.5-17 MHz). Patients with in situ disease were not subject to axillary assessment/surgery. Lymph nodes with cortical thickness of 3 mm or more, irregular margins, and effacement of the fatty hilum were considered suspicious and were graded A3 and above. All these patients were subjected to US‑guided NCB. Patients graded A1/A2 and those with biopsy-proven benign nodes had sentinel node biopsy. Patients with positive biopsy for metastatic disease were subjected to axillary lymph node dissection (ALND).

Results

Axillary assessment for patients graded A3 and above (Group B) met or exceeded NHS standards for sensitivity (0.93), specificity (0.92), and PPV (0.92). NPV (0.92) was slightly below NHS standard. The lower performance metrics in Group B (A1-5) fell short of NHS guidelines in all four metrics, highlighting the need for potential improvements in the diagnostic process or the use of additional diagnostic tools to enhance accuracy.

Conclusion

Axillary assessment in A3 patients met or exceeded NHS standards, with sensitivity, specificity, and PPV at 0.92, though NPV was slightly lower. A1/A2 patients underperformed across all measures. The findings support AUS as a reliable tool for breast cancer staging and management.

## Introduction

Breast cancer remains the leading cancer among women globally, with approximately 2.3 million new cases reported in 2022, underscoring the need for effective diagnostic and therapeutic strategies to improve patient outcomes [1]. Accurate assessment and staging of axillary lymph nodes (LNs) is essential, as these nodes are often the first site of metastasis. Their status informs treatment decisions, such as surgery, chemotherapy, or radiation, and helps guide prognosis and planning [1,2].

Historically, axillary lymph node dissection (ALND) was the standard for staging and managing the axilla in invasive breast cancer. However, with the widespread adoption of sentinel lymph node biopsy (SLNB) in the late 1990s, ALND has shifted toward a role focused on local control in patients with confirmed nodal disease. Axillary ultrasonography (AUS) has since emerged as a key non-invasive tool in preoperative staging, enabling the detection of suspicious LNs and guiding targeted needle biopsies. This approach enhances diagnostic accuracy, reduces unnecessary surgery, and supports SLNB as the preferred method in clinically node-negative patients [3,4].

AUS can identify metastatic disease based on defined morphological criteria, allowing US-guided needle biopsies of suspicious nodes [3]. Evidence suggests that nodal disease detected via AUS may carry a higher axillary burden than that identified through SLNB alone, which has implications for selecting candidates for ALND or targeted axillary dissection (TAD).

The National Institute for Health and Care Excellence (NICE) guidelines recommend pre-treatment AUS in patients with early and locally advanced invasive breast cancer [4]. If abnormal nodes are detected, needle sampling is advised to confirm metastasis, reinforcing the role of AUS in guiding clinical decisions [4,5].

Despite its advantages, the accuracy of AUS can vary due to factors such as operator experience, patient characteristics (e.g., breast density, body habitus, BMI, tumour location, prior surgery), and equipment quality, as highlighted in the NAUTILUS trial by Chang et al. [6]. Sensitivity, specificity, negative predictive value (NPV), and positive predictive value (PPV) are key metrics for evaluating its performance [7].

This study investigates the clinical utility of combining AUS with US-guided needle core biopsy (NCB) as a comprehensive staging strategy. It analyses the sensitivity and specificity of this approach in patients with breast cancer, comparing findings to national benchmarks. Axillary assessment in patients graded A3 and above met or exceeded NHS standards for sensitivity (93% vs ≥50%), specificity (92% vs ≥95%), and PPV (92% vs ≥70%), while NPV (92%) fell slightly below the national standard of ≥95% [7]. By evaluating the implications and outcomes of preoperative axillary staging, this research aims to deepen understanding of AUS’s role in breast cancer management, optimize staging protocols, and audit the performance of a single-unit AUS service against national standards [7].

Aims and objectives

This study aimed to evaluate the diagnostic accuracy of preoperative AUS combined with US‑guided NCB in patients with invasive breast cancer at Leighton Hospital, benchmarked against national NHS standards. The primary objective was to determine the sensitivity, specificity, PPV, and NPV of AUS and NCB in detecting nodal metastasis. A secondary objective was to examine concordance between preoperative imaging findings and final histopathology.

## Materials and methods

Study design and study cohort

This retrospective observational study was carried out at the Breast Unit of Leighton Hospital, part of the Mid Cheshire Hospital Trust (MCHT). It included all individuals assessed, diagnosed, and treated for breast cancer between January 1 and December 31, 2022. Of the 299 patients initially identified, one case of lymphoma was excluded, yielding a final sample size of 298.

Inclusion Criteria

Patients diagnosed with early-stage breast cancer (Stage I-IIIA) who underwent assessment and treatment at the Breast Unit, Leighton Hospital, between January 1, 2022, and December 31, 2022.

Exclusion Criteria

Patients with locally advanced, distant metastatic, or recurrent breast cancer and those identified with primary LN disease like lymphoma were excluded.

All patients who met the inclusion criteria within the specified timeframe were included in the study. The patients were initially evaluated by clinical examination, mammography, and breast ultrasound, according to the current NHS guidelines. AUS was performed using a high-frequency linear array transducer (7.5-17 MHz). During the procedure, the patients were positioned in a supine oblique position with the arm abducted and externally rotated. At least one image of the most suspicious LN (in the longitudinal direction) was saved digitally. The assessment was performed by two consultant radiologists and one consultant radiographer using standard techniques. Inter-observer variation remains one of the limitation of the study. LNs were categorized from A1 to A5 based on US evaluation where A1 was normal, A2 was benign, A3 was indeterminate, A4 was suspicious for malignancy and A5 was highly suspicious for malignancy. According to institutional guidelines at the time of the study, a biopsy of a suspicious LN was performed in cases of sonographically “A3” nodal status. Nodes with cortical hypertrophy > 3 mm with eccentric/ focal cortical hypertrophy, round in shape, and loss of the central hilum were considered A3 and above and biopsied.

Because cortical hypertrophy could not be measured in some cases (due to full metastatic affection of the LN), a combined variable (“No Hilum”) was set up combining both variables, i.e., corresponding to either a “loss of the central hilum” or an “eccentric cortical hypertrophy ≥3 mm”. In cases where an inter-observer discrepancy was observed, the case was reviewed by another experienced consultant radiographer/ radiologist.

Surgical approaches to the axilla

All included patients were treated according to current national and international guidelines based on multidisciplinary in-house meeting decisions. Patients who had either non-suspicious LN after AUS or proof of benign LN on histopathology after AUS-guided needle biopsy were graded as A1 and A2 on AUS and underwent SLNB, performed using a dual technique with blue dye and Magtrace (Endomag, Cambridge, UK), a superparamagnetic iron oxide tracer approved by NICE for sentinel LN localization in breast cancer surgery. For patients with no evidence of blue dye or poor/nil Magtrace signals, axillary four-node sampling was performed.

In patients undergoing neoadjuvant chemotherapy (NACT), AUS was employed post-treatment to assess nodal response. Individuals with biopsy-confirmed axillary LN metastases at initial presentation proceeded to ALND following completion of NACT. The excised LNs were subjected to histopathological evaluation to quantify residual tumour burden and identify regressive changes, such as fibrosis and residual malignant cells, indicative of prior metastatic involvement. These histopathological findings were categorized as complete pathological response (CPR), defined by the absence of residual viable tumour cells and presence of regressive changes such as fibrosis and histiocytic infiltration; partial pathological response (PPR), characterized by residual tumour cells alongside evidence of treatment-induced regression; or no response, indicating persistent viable tumour cells with minimal or absent regressive features (Table 1). Conversely, patients without clinical or radiological evidence of axillary nodal disease at baseline underwent SLNB as the preferred staging procedure.

**Table 1:** Patient demographics and cancer characteristics DCIS: ductal carcinoma in situ, CPR: complete pathological response, PPR: partial pathological response

Clinical Characteristics	Diagnostic and Pathological Categories	Measured Values	
Demographics	Mean Age	61.3 (30-91)	
	Mean Tumour size	44.01mm (0-240mm)	
Source	Screening	146 (48.99%)	
	Symptomatic	143 (48.65%)	
	Follow-up Mammogram	8 (2.68%)	
	Incidental finding on CT	1 90.3355)	
Disease	Invasive Ductal Cancer (IDC)	212 (71.14%)	
	Invasive Lobular Cancer (ILC)	33 (11.07%)	
	Mixed (IDC/ILC)	6 (2.013%)	
	Others	11 (3.69%)	
	DCIS only	36 (12.08%)	
T stage	T1	171 (57.38%)	
	T2	80 (26.84%)	
	T3	9 (3.69%)	
	T4	2 (0.67%)	
N stage	N0	215 (72.14%)	
	N1	38 (12.75%)	
	N2	10 (3.355%)	
Neoadjuvant chemotherapy	26 (8.72%)	CPR	11 (42%)
		PPR	12 (46%)
		No response	3 (12%)

The patients diagnosed with ductal carcinoma in situ (DCIS) did not undergo axillary surgery. However on initial evaluation, AUS was performed as standard practice in the unit. However, those identified as having invasive or microinvasive disease on final histology were subjected to SLNB as a second operation.

Pathologic evaluation as reference results

The LNs retrieved during ALND or SLNB were formalin-fixed, embedded in paraffin, sectioned, stained, and processed according to the current guidelines. Immunohistochemistry was performed in selected cases with an unclear histopathology. The presence of parenchymal or subcapsular macro metastases (≥2 mm), micro metastases (>0.2 mm but <2 mm), or isolated tumour cells (≤0.2 mm) was assessed and categorized accordingly.

Data collection

Data was collected for post-operative results for primary tumours and axillary surgery, including SLNB, axillary sampling, and ALND. Data were recorded in Microsoft Excel (Redmond, WA, USA) and secured on a password-protected shared drive, accessible to team members. All cases were discussed pre- and post-operatively in the Breast Multidisciplinary meeting, and outcomes were recorded using the cancer registry software SOMERSET. 

Quality assurance

AUS and core biopsies were performed by experienced breast radiologists using high-frequency transducers and standardised protocols. Suspicious nodes were sampled with 14G needles, and results were reviewed in multidisciplinary team meetings. Equipment was regularly maintained, and diagnostic performance was monitored through standard practice and procedures and by performing local audits.

Statistical analysis

Descriptive statistics and standard diagnostic accuracy measures were used to evaluate the performance of AUS. These metrics included sensitivity, specificity, NPV, PPV, the diagnostic odds ratio (DOR), and the Youden Index (YI) to determine how accurately AUS performs in routine clinical practice. The DOR reflects the overall effectiveness of a diagnostic test by comparing the odds of a true‑positive result with the odds of a false‑negative result. The YI incorporates both sensitivity and specificity, with values ranging from 0 (no diagnostic usefulness) to 1 (perfect discrimination). Accuracy was defined as the proportion of correct classifications, including both true positives and true negatives, among all evaluated cases. The initial AUS assessment (index test) was compared with the final pathological reference standards, including ALND, SLNB, or positive CNB findings.

## Results

**Figure 1:**
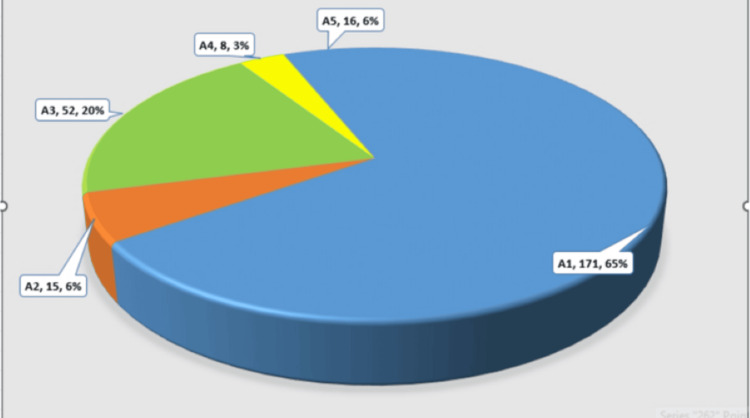
Total number of patients subjected to axillary ultrasound, n=262

In total, 298 patients were included in this study. The mean age at diagnosis was 61.3 ± 15.3 years (range 30-91). Approximately half of the patients (n=144, 48.32%) presented with symptoms, with the remaining half from screening (n=145, 48.65%) and a small proportion from follow-up mammograms and incidental findings on other scans (n=9, 3.0%). Most carcinomas were invasive-ductal (n=212, 71.14%) and of histologic tumour grade 2 (n=165, 55.36%). The mean tumour size was 44.0 ± 60.0 mm (range 0-240). After being diagnosed with DCIS (n=36, 12.08%), during further assessment, no further axillary examination/biopsy was performed. Among all those patients identified with invasive disease (n=262, 87.91%), 185 (70.61%) belonged to the group of patients with HR+/HER2 negative disease, 39 (14.88%) were HER2+, and 38 (14.50%) belonged to triple negative disease group. Table 1 shows detailed clinical and pathological characteristics of the study cohort. On routine AUS for invasive disease, 210 axillae (70.60%) were classified as sonographically node-negative (AUS-), whereas 76 patients (29.00%) were diagnosed as sonographically node-positive on AUS assessment (AUS+). Among 298 patients, axillary assessment was completed in 262 (87.91%) patients (Figure 1). Among these, on Breast Imaging-Reporting and Data System (BI-RADs) scoring, 171 (57.38%) patients were labelled as A1, 15 (5.03%) as A2, 52 (17.44%) as A3, eight (2.68%) as A4, and 16 (5.36%) as A5. In total, 77 (25.83%) patients with score A3 and above underwent US-guided NCB. For nodes graded as A3, mean cortical thickness was 3.72 ± 0.88 mm (range 2.7-6.2). A4 had a mean of 6.28 ± 1.30 mm (range 4-9.2), and A5 had a mean of 10.42 ± 7.45 mm (range 3.2-33). One patient graded as A2 was subjected to NCB based on clinical suspicion and was found to be positive; hence, ALND was planned. Based on the biopsy results, four were graded as B1 (insufficient material and biopsy was repeated in one), 34 were B2 (benign), and 43 were B5 (positive nodes). Based on these results, 216 patients had SLNB (breakdown: A1, 167; A2, 14; A3, 32; A4, 2) and 43 had ALND (all A5, six scored as A4, 20 from the A3 group, and one patient labelled as A2). Three patients had axillary sampling, as no signals were identified from dual tracers. No axillary procedure was performed in 36 patients, as they were diagnosed with DCIS on pre-operative biopsy. However, post-operatively, 10 patients were diagnosed with small invasive cancers and eventually underwent SLNB, which were all negative.

Interpreting the results of axillary surgery

**Figure 2:**
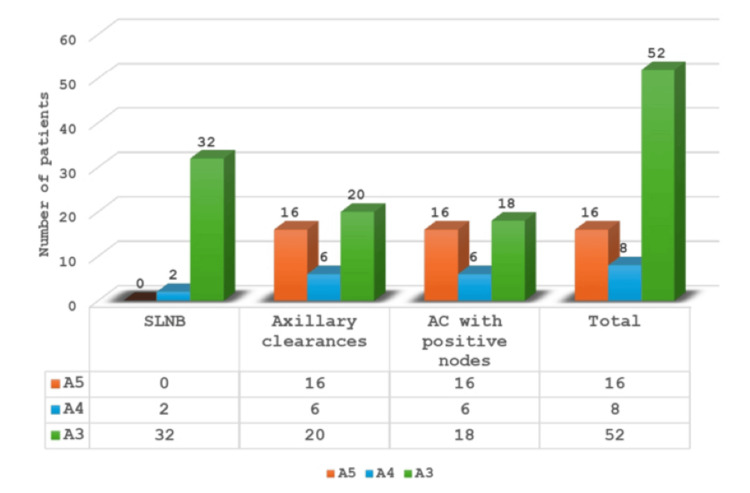
Axillary surgery based on axillary assessment (A1-A5) SLNB: sentinel lymph node biopsy, AC: axillary clearance

Patients (n=171) were labelled A1 on BI-RADs scoring and underwent SLNB and axillary sampling and eight patients were found to have positive nodes and underwent ALND as a secondary procedure. Among these, four patients had invasive lobular cancer. Patients with A2 assessment pre-operatively and two with positive SLNB underwent ALND as a secondary procedure. Among the A3 group, 32 SLNBs were performed, three were positive, two underwent ALND as a secondary procedure, and one had axillary radiotherapy (XRT). Among those who had axillary clearance (AC), 18 were identified with positive nodes and no positive nodes were identified in two patients. In the A4 group, two had SLNB which was negative, and six underwent AC, all showing positive nodes. In the A5 group, 16 patients had AC, all of which showed positive nodes (Figure 2). Axillary clearance was performed as a secondary procedure for 13 patients who were first subjected to SLNB and identified with positive nodes.

Performance of AUS in routine clinical assessment for breast cancers

Two groups were analysed for diagnostic performance. Group 1 included patients with BI‑RADs scores of A3 and above (n = 77). In this group, AUS combined with needle core biopsy had a sensitivity of 93.3%, a specificity of 92.7%, a NPV of 92.0%, and a PPV of 93.0%. The YI, which is a summary measure that combines sensitivity and specificity into a single value, was 0.83 (95% CI). A YI close to 1 indicates excellent balance between correctly identifying true positives and true negatives, showing that the test is highly effective. The DOR, which expresses how much greater the odds are of a positive test in patients with disease compared to those without, was 121.83. A high DOR reflects very strong discriminatory power of the test.

Group 2 included all patients assessed with axillary ultrasound (n=262). In this group, sensitivity was 77.01%, specificity was 72.7%, NPV was 72.0%, and PPV was 77.0%. The YI was 0.54 (95% CI), which indicates a moderate balance between sensitivity and specificity, meaning the test is less effective at correctly identifying both true positives and true negatives compared to Group 1. The DOR was 11.73, which reflects moderate diagnostic accuracy and a weaker association between test results and disease status compared to Group 1.

LNs with a cortical thickness of ≥3 mm (n=76) and with features such as round shape, or loss of the central hilum were found significantly more frequently in malignant than in benign axillae (p < 0.001). The p-values for the A1 and A2 groups were greater than 0.05, suggesting no statistically significant difference between these groups. However, the p-values for A3 and the above groups were much less than 0.05, indicating a statistically significant difference. This also indicates that the criterion “cortical hypertrophy ≥3 mm” showed the highest sensitivity for the detection of axillary LN metastasis on AUS. At the same time, a cortex diameter <3 mm had the lowest specificity for excluding axillary metastasis. Overall, a high DOR indicates that AUS is very effective in distinguishing between patients with and without positive LNs in this group. A higher DOR suggests a strong association between the test results and the confirmed status of LNs on final histology. The YI of 0.83 reflects a good balance between sensitivity and specificity, meaning that the test is both good at identifying true positives and true negatives. These metrics suggest that AUS is a reliable diagnostic tool for patients with scores of A3 and above, making it a valuable part of the diagnostic process for these patients. However, when compared with group 2 (A1-A5), a lower DOR implies moderate diagnostic accuracy in all patients. Similarly, the YI of 0.54 suggests a moderate balance between sensitivity and specificity. This means that the test is less effective at correctly identifying both true positives and true negatives than the first group.

## Discussion

AUS is a crucial imaging technique for assessing the axillary nodal status in patients with newly diagnosed breast cancer. It is particularly effective in identifying metastatic disease in axillary LNs based on specific morphological criteria such as cortical thickness, shape, and the presence or absence of the hilum [7,8].

A meta-analysis by Houssami et al. (2011) reported a pooled sensitivity of 61.4% and specificity of 82.0% [9]. This variability is due to differences in operator experience, patient characteristics, and quality of ultrasound equipment. Combining AUS with fine-needle aspiration biopsy (FNAB) increases sensitivity to 79% and specificity to 98%, highlighting the importance of operator skill and the benefits of combining US with biopsy techniques [8-10]. Axillary assessment in patients with LNs graded A3 and above is an effective and reliable method [11]. Ultrasound‑guided biopsy is widely used for the evaluation of axillary LNs and is recognized for its high specificity, often reported around 91.6%, indicating strong reliability when metastatic disease is detected. However, its sensitivity is considerably more variable, with published studies reporting values as low as 52.25%, reflecting a substantial false‑negative rate [12,13].

A high NPV (80% to 95%) indicates that a negative US result reliably excludes LN metastasis, reducing the need for invasive procedures. PPV varies from 56% to 90%, emphasizing the need for confirmatory biopsies of suspicious nodes to ensure accurate diagnosis and treatment planning [10].

In our study, the high sensitivity (0.93) indicates that the axillary assessment correctly identified 93% of patients with positive nodes. A specificity of 0.92, slightly above the NHS standard, indicates that 92% of patients without positive nodes were correctly identified. This reduces the likelihood of false positives, which can lead to unnecessary treatments and the associated patient anxiety. Similarly, the NPV of 0.92 suggests that 92% of patients predicted to not have positive nodes indeed did not have them. Although this is slightly below the NHS standard of 0.95, it still indicates a high probability that a negative test result will be accurate. A high NPV is essential for reassuring patients and clinicians that negative results are reliable. A PPV of 0.93, higher than the NHS standard, indicates that 93% of patients predicted to have positive nodes actually had them. The findings for this subgroup are consistent with those in the existing literature, which emphasizes the importance of high sensitivity and specificity in diagnostic tests. For instance, findings from other studies and a systematic review reported earlier that US-guided biopsy had a sensitivity of >50% and specificity of >90% for detecting axillary LN metastasis. The higher sensitivity observed in these studies suggests that the axillary assessment for patients with axillary assessment scores of A3 and above is particularly effective, potentially due to the targeted nature of the subgroup [12,14].

However, when the analysis was expanded to include all patients who underwent AUS (A1-A5), the overall diagnostic performance decreased, yielding a sensitivity of 0.77, specificity of 0.72, NPV of 0.72, and PPV of 0.77. These results fall short of the NHS standard of 0.85 [4]. Similar to the case with specificity of 0.72, the NPV of 0.72 is significantly below the NHS standard of 0.95, indicating a higher likelihood of false negatives. Improving the NPV is important for providing reliable reassurance to patients and clinicians. A PPV of 0.77, close to the NHS standard, indicates that 77% of patients predicted to have positive nodes actually had them [15-18]. These findings are consistent with studies in the literature showing that the sensitivity and specificity of US-guided biopsy can vary widely, depending on the patient population and the criteria used for assessment. This could also imply that in patients A3 and above, where US-guided NCB was deployed, findings were more in line with those reported in the literature and guidelines.

Study limitations

This study is retrospective in nature, which inherently presents certain design constraints, including potential selection bias and reliance on pre-existing medical records rather than prospective data collection. AUS assessments were conducted solely by a single consultant radiographer or radiologist, without cross-referencing or independent verification, which may introduce observer bias and limit the generalizability of findings. Additionally, a fixed threshold of 3mm was used as the cut-off value for LN cortical thickness, which may not account for variations in nodal characteristics across different patient populations and could affect diagnostic sensitivity and specificity.

## Conclusions

The high diagnostic accuracy in the A3 and above subgroups underscores the effectiveness of axillary assessment in patients with breast cancer, validating its role as a reliable diagnostic tool. Focusing on patients with higher BI-RADS scores can improve the reliability of US-guided biopsies, leading to better patient outcomes. The lower accuracy in the broader group suggests the need for refining the assessment criteria and incorporating additional diagnostic methods to enhance the overall accuracy. For instance, combining AUS with other imaging modalities, such as magnetic resonance imaging (MRI) or positron emission tomography (PET), may enhance the diagnostic accuracy.
